# Unilateral Madarosis Revealing Blepharitis Caused by Microsporum audouinii: A Case Report

**DOI:** 10.7759/cureus.78429

**Published:** 2025-02-03

**Authors:** Mouhib Loubna, Oumaima Dahreddine, Mehdi Khamaily, Amine Razzak, Mohamed Elbelhadji

**Affiliations:** 1 Department of Ophthalmology, Faculty of Medicine, Mohammed VI University of Health Sciences (UM6SS), Casablanca, MAR

**Keywords:** blepharitis, dermatophytes, madarosis, microbiological examination, microsporum audouinii

## Abstract

Madarosis, defined as partial or total loss of eyelashes, is an unusual symptom that can reveal a variety of underlying conditions. We report a rare case of unilateral madarosis associated with blepharitis caused by Microsporum audouinii, a dermatophyte responsible for ringworms. An eight-year-old boy presented with ocular pruritus with erythematous squamous lesions of the right eyelid evolving for four days. An initial diagnosis of eczematous blepharitis was made, and symptomatic treatment was initiated. However, the evolution was unfavorable, marked by the appearance of madarosis.

The patient's clinical examination revealed chronic mixed blepharitis, while his sister had scalp ringworm. Mycological examination of the palpebral scales and eyelashes revealed Microsporum audouinii, leading to the diagnosis of blepharitis secondary to dermatophytosis. We initiated an appropriate antifungal treatment, both systemic and topical, resulting in favorable clinical evolution. We noticed a progressive regrowth of the eyelashes and complete resolution of the palpebral lesions. This case illustrates the importance of considering infectious etiologies, including fungal infections in the differential diagnosis of atypical blepharitis resistant to treatment, indicating the importance of a microbiological examination. An early and appropriate treatment can prevent chronic complications, particularly madarosis.

## Introduction

Blepharitis is a chronic inflammation of the eyelids that can profoundly affect the patient's well-being. It is generally bilateral and symmetrical. Its symptoms include redness, itching, and crusting [1]. The most common causes of blepharitis are bacterial infections, particularly staphylococcal species, and infestations by Demodex mites [2,3]. 

Madarosis refers to the loss of eyebrows or eyelashes. This clinical sign occurs in various diseases ranging from local dermatological disorders to complex systemic diseases. It can be associated with blepharitis [4].

Dermatophytoses are fungal infections caused by dermatophytes, fungi that infect keratinized tissues such as the skin, hair, and nails. These infections are commonly known as ‘ringworms’ and can take a variety of forms [4,5].

While bacterial infections and Demodex mites are more commonly associated with blepharitis, dermatophytes can be one of the rare causes of chronic blepharitis.

In this paper, we report the rare case of a child with unilateral madarosis revealing blepharitis caused by Microsporum audouinii.

## Case presentation

An eight-year-old child presented with pruritic right palpebral lesions with photophobia and sand grain sensation evolving for four days. He was initially diagnosed with periocular eczema and received a one-month local treatment on the right eye, consisting of antibiotics combined with tobramycin/dexamethasone corticosteroids and antihistamines. The evolution was unfavorable, with an onset of madarosis. Further investigation revealed a recent history of scalp ringworm in the older sister.

**Figure 1:**
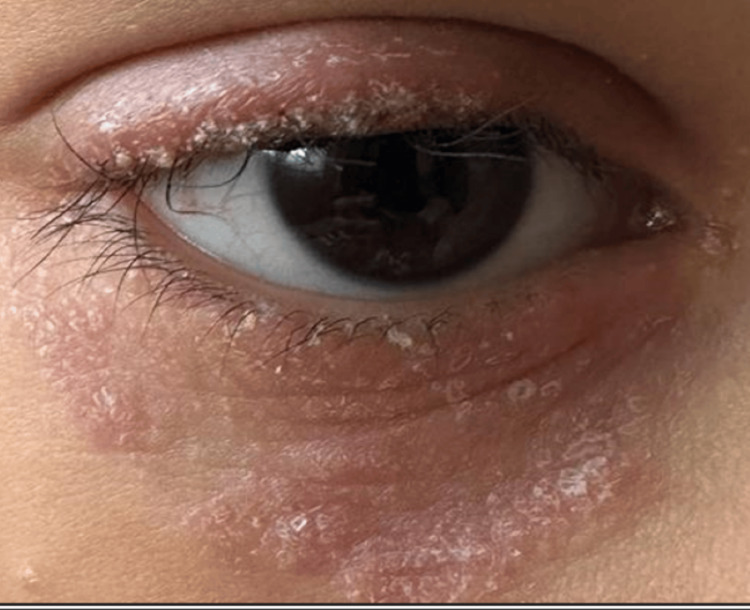
Anterior eczematous blepharitis of the upper and lower eyelids associated with madarosis

Clinical examination revealed extensive erythematous and scaly palpebral lesions in the right eye. Best corrected visual acuity was 20/20 in both eyes. The slit-lamp examination revealed madarosis and mixed blepharitis with telangiectasias at the free edge and numerous crusty plaques in the right eye (Figure 1). The fluorescein test was positive on the right, with superficial punctate keratitis predominating in the upper region. The rest of the ophthalmological examination was unremarkable. Dermatological examination revealed no other lesions or alopecia.

**Figure 2:**
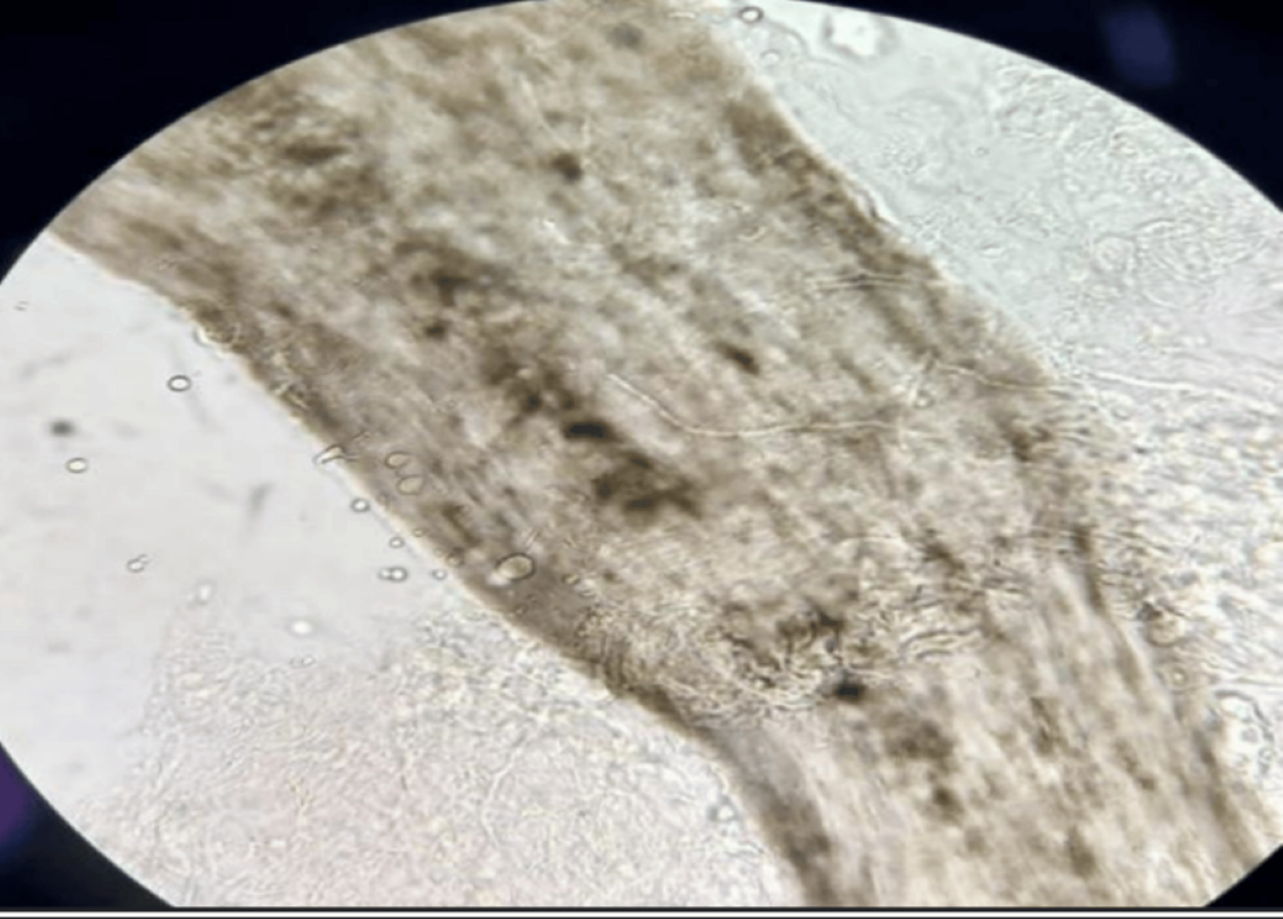
Endothrix microsporum cilial parasitism (magnification x40 x10)

**Figure 3:**
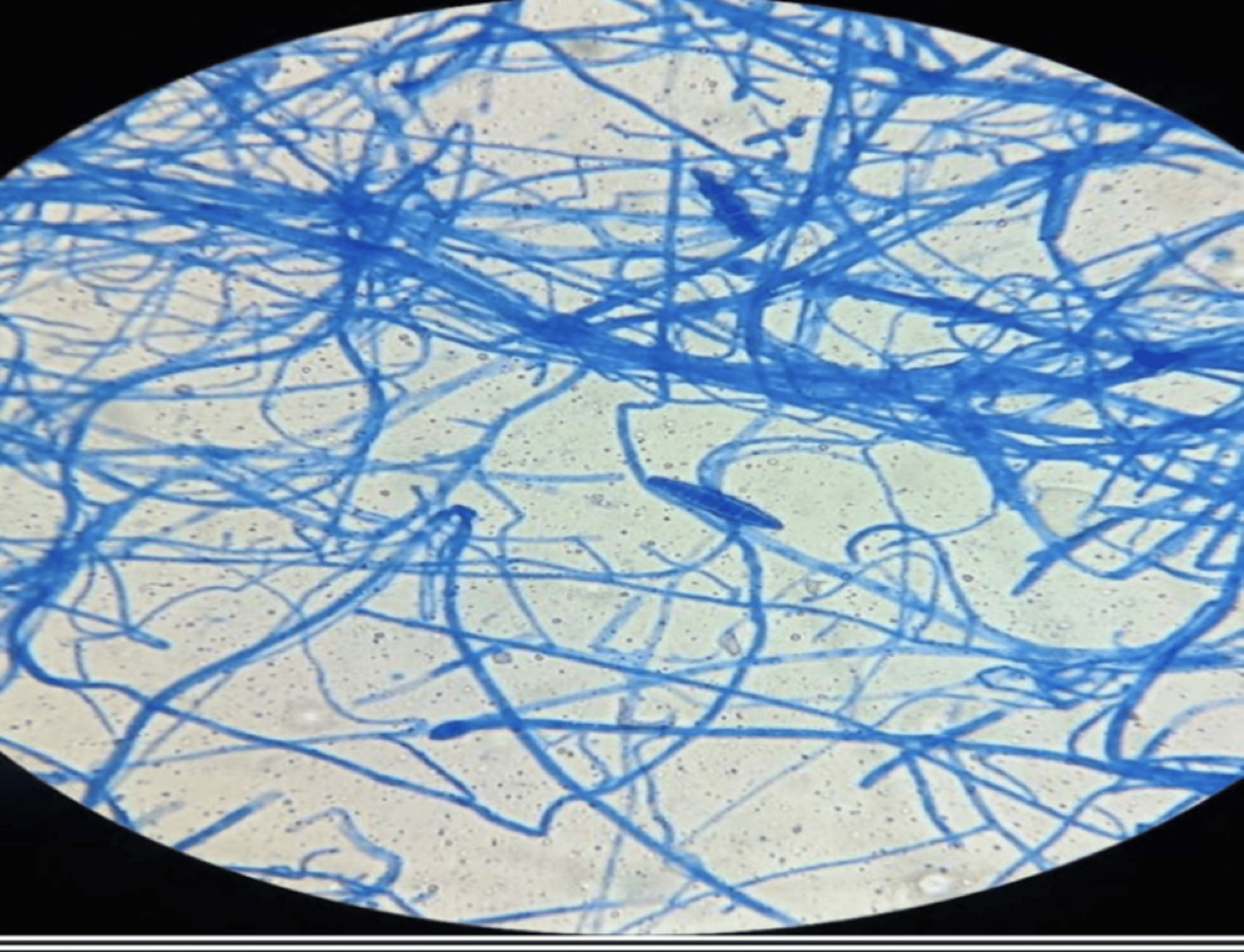
Spores and racquet-shaped hyphae (lactophenol blue staining)

Mycological examination of the eyelashes was performed. Direct examination (using a 10% solution of potassium hydroxide) revealed endothrix mycelial filaments (Figure 2) associated with the germ Microsporum audouinii and the presence of spores and racquet-shaped hyphae (Figure 3). Systemic treatment was initiated with griseofulvin at a dose of 500 mg/d for six weeks and amoxicillin-clavulanic acid as bacterial superinfection was suspected. He also received local treatment with quinolone eye drops and ointment, artificial tears, and terbinafine cream for external use and recommendations on good eyelid hygiene.

**Figure 4:**
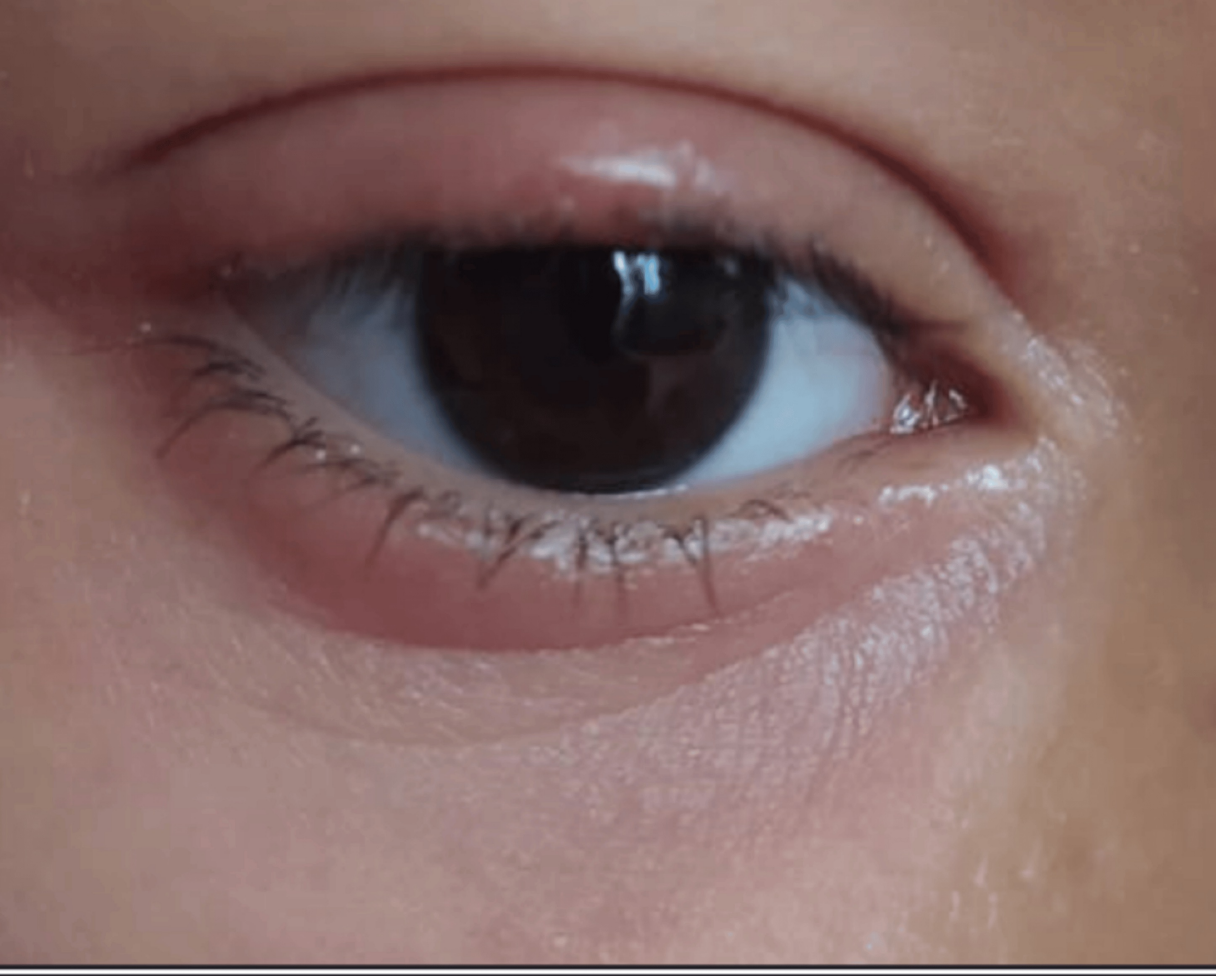
After two weeks of treatment: regrowth of eyelashes and significant improvement of eyelid lesions

**Figure 5:**
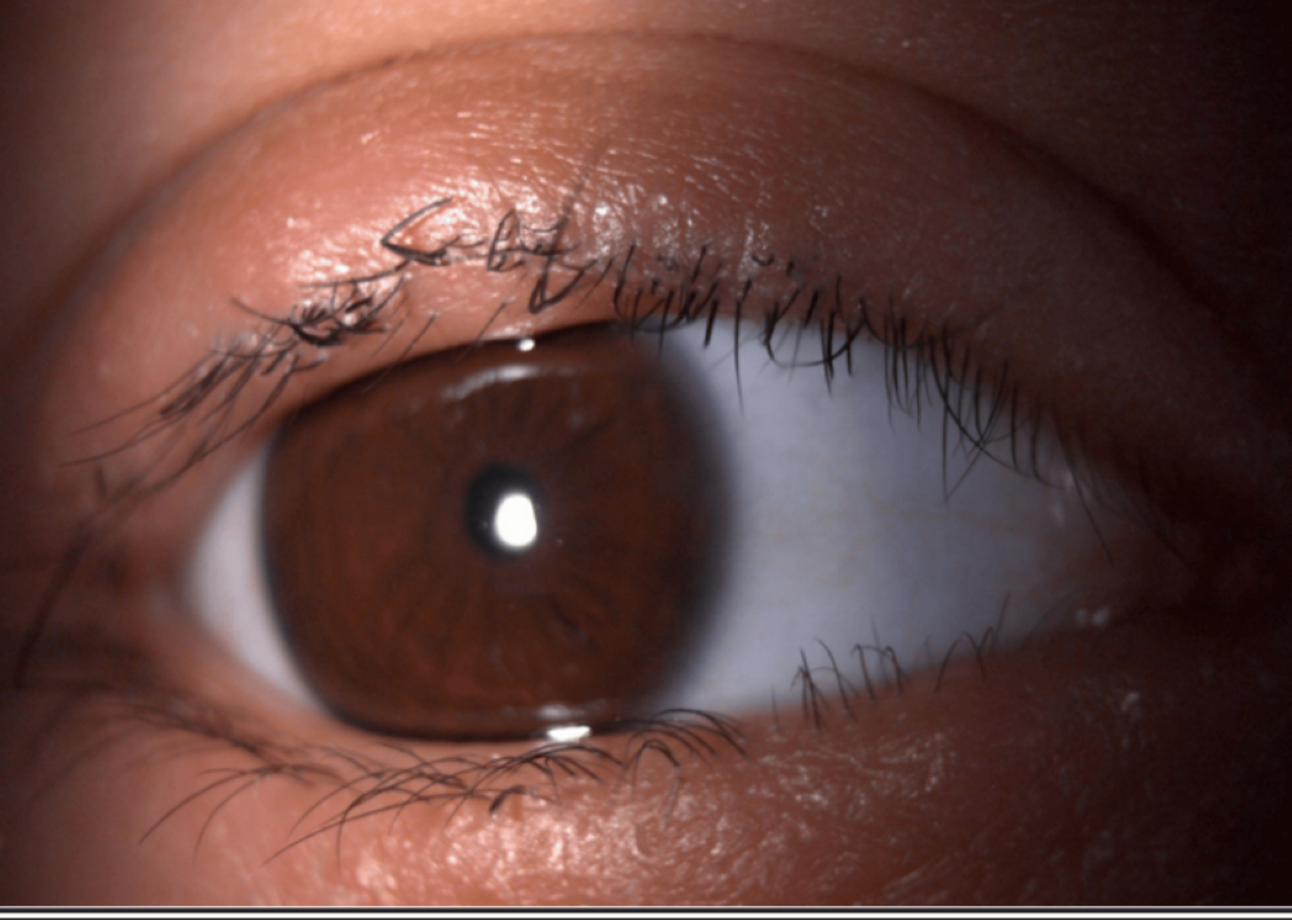
Appearance after one-month treatment: persistence of mild edema and complete regrowth of eyelashes

The clinical course was favorable showing a regrowth of eyelashes and a noticeable improvement in palpebral lesions after two weeks of treatment, with complete regrowth of eyelashes observed after one month of treatment (Figures 4, 5).

## Discussion

Dermatophytosis is a disease caused by keratinolytic fungi that mainly affects the skin and appendages. These fungi have a worldwide distribution and can occur in the form of epidemics [6]. Microsporum audouinii predominates in Africa and Asia, with few recent outbreaks in Europe and America. It is mainly responsible for ringworms of the scalp with large patches of alopecia in children and cutaneous mycosis. Transmission is mainly anthropophilic, either by direct contact or via contaminated objects. Family transmission, as observed in this case with ringworm in the patient's sister, is a well-documented risk factor [7,8].

The presence of unilateral madarosis should prompt a search for underlying pathologies such as chronic bacterial or fungal infections, autoimmune disorders such as systemic lupus erythematosus, or tumors such as basal cell carcinoma [9]. 

In this case, the unilateral nature of the blepharitis and its resistance to conventional treatment prompted a mycological examination of the eyelashes. Due to the highly contagious nature of this dermatophyte, a systematic examination of contact cases, particularly the family, is necessary.

In the literature, there are only a few cases of blepharitis linked to dermatophytes; these dermatophytes include Trichophyton benhamiae, Trichophyton tonsurans, and Trichophyton verrucosum. Trichophyton benhamiae was identified in a 13-year-old girl who had right eyelid swelling and eyelash loss for two weeks. The lesions were presented as erythematous patches with scales and tiny pustules on the right upper and lower eyelids with broken eyelashes. Microscopic examination of broken eyelashes demonstrated many chains of arthroconidia and hyaline hyphae in an endothrix invasion pattern. Fungal cultures of right eyelid scales, eyelashes, and right thigh lesions all grew Trichophyton benhamiae, which was diagnosed by both morphological characters and sequencing of the internal transcribed spacer region of the ribosomal DNA [10]. Trichophyton tonsurans caused blepharoconjunctivitis in a previously healthy eight-year-old boy [11]. Trichophyton verrucosum caused chronic blepharitis in a 40-year-old woman for four years [12].

There are even fewer papers specifically linking blepharitis to Microsporum audouinii. We found one particular paper by Sahin et al. describing a 69-year-old man with a four-year history of blepharitis caused by Microsporum isolated in culture [12]. This shows the distinctive nature of our paper.

This raises the question: Is this rarity due to the infrequent occurrence of dermatophyte-related blepharitis or is it because investigating the etiology of blepharitis, particularly through microbiological sampling is not a common practice?

## Conclusions

Blepharitis, an inflammation of the eyelids, can potentially alter a patient’s quality of life. Treatment is usually based on eyelid hygiene, which can be combined with antibiotic therapy, anti-inflammatories, or treatment of corneal complications. In our practice, we came across a number of cases that are resistant to treatment. It is therefore necessary to look more frequently for other etiologies.

Although dermatophytes are a rare etiology of blepharitis, our case highlights the importance of considering fungal infections as a differential diagnosis for atypical or even typical but treatment-refractory blepharitis, indicating the value of a well-documented patient's history and microbiological examination. Larger controlled studies should be carried out to further validate the link between blepharitis and dermatophytes.
